# Supplementary material for: Learning curve and surgical outcome of robotic assisted colorectal surgery with ERAS program
Source: Sci Rep. 2022 Nov 29;12:20566. doi: 10.1038/s41598-022-24665-w (PMC9709162; doi:10.1038/s41598-022-24665-w)
Supplement: Supplementary file 1 — Supplementary Information 1. [file 41598_2022_24665_MOESM1_ESM.zip › LSC Raw data-Ñ[▒K/067/H_ñw╝╨░O▒Kñσ.pdf]

正式摘要(電子簽章：000000187039206)

W52 / W52 ☐ 英文標題(注意:無英文姓名-請補病人基本檔!) ☐ 【讀取】憑證

臺中榮民總醫院

出院病歷摘要

[redacted]

(9)入院日期	109/02/12 (2020) 直腸外科 W52 - 112
(10)轉科日期	
(11)出院日期	109/03/04 (2020) 住院天數計 21 日

診斷

- 入院 :1.Ascending colon cancer
  2.AMI, inferior wall, post PCI wtih stent at Chen-Chin H 11/30 2009
- 出院 :1.Adenocarcinoma of ascending colon, s/p laparoscopic right hemicolectomy on 2020/2/14, pT3N0, stage IIA
  2.AMI, inferior wall, post PCI with stent on 2009/11/30

主訴

- Incidental finding of ascending colon cancer

病史

- This is a 69 year old male patient with history of
  1. AMI, inferior wall, post PCI wtih stent at Chen-Chin H 11/30 2009
  According to the patient, he had health examination and found occult blood positive. He recieved colonoscopy later and was diagnosed of Ascending colon cancer. He came to our CRS OPD for 2nd opinion. This time, he was admitted to our ward for schedule Lapa Right hemicolecotmy.

體檢發現

- General appearance :
  - easy looking , conscious clear , without cardiopulmonary distress
  - Vital signs自動帶入date 體溫(BT) 脈搏(P) 呼吸(R) 血壓(BP) 身高(BH) 體重(BW) BMI
    20200212 36.1℃ 81次/分 18次/分 140/77mmHg 166.7cm 79.8kg 28.7
- HEENT :
  - Eyes: no anemic conjunctiva, no icteric sclera, normal EOM
  - ENT: no otorrhea, no rhinorrhea, no erythematous mucosa, no injected tonsil, no deformity
- Neck: supple, freely movable, trachea midline,no deformity, no engorged jugular vein , no palpable thyroid or lymph node enlargement
- Chest:
  - Inspection: Grossly normal, symmetric and fully expansion, no accessory muscle used, no operation scar, no spider angiomata, no gynecomastia
  - Palpation: no palpable mass or nodule, no subcutaneous emphysema, no tenderness
  - Ausculation: clear breathing sound
- Heart:
  - Percussion: normal heart size
  - Ausculation: regular heart beat, normal S1, S2, no gallop, no murmur
- Abdomen:
  - Inspection: in
    - flat contour, symetrically, no scar, no stria, no dilated veins, no rashes
  - Ausculation: normoactive bowel sound, no abdominal bruits
  - palpation: soft, no muscle guarding or rigidity, no local tenderness, no rebounding pain, no palpable mass, no palpable pulsation
  - Percussion: no shifting dulllness, no hepatomegaly, no splenomagaly
-

Peripheral pulsation :	CA	BA	RA	FA	PA	PTA	DPA
Right side :	++	++	++	++	++	++	++/+++
Left side :	++	++	++	++	++	++	++/+++

- Genito-anus: no tenderness or palpable mass lesion or rectal shelf, normal external genitalia, normal anal tone, brown stool

CRS CONDITION

-
  - Lung

手術日期及方法

- 手術日期及方法20200214 Laparoscopic right colectomy and anastomosis

住院治療經過

- After admission, pre-operation survey was done and no contraindication was noted. Laparoscopic right colectomy was performed smoothly. Cefazolin+Metronidazole were given as antibiotic treatment. We removed NG tube on POD1. The patient recovered very slowly. The patient suffered from abdominal distension and nausea. The patient can only tolerated intermittent diet. 2/27 Abdominal CT revealed few fluid accumulation over bilateral paracolic gutter and the pelvis. We removed penrose drain after that. Then he had flatus and stool passage afterward. He started to try oral diet gradually without discomfort, under stable condition, we arranged MBD and OPD f/u for him.

合併症

一般檢查

- 累積報告 :

DATE	TIME	ALB	TP	BIL.T	BIL.D	ALKP	AST	ALT	LDH	CHOL	TG	HDL-C	LDL	UA	NH3	AMY
1090212	2003		6.6							128	78	37	72	6.4		
1090214	1841	3.7		1.6	0.4		16	12								
1090217	0936	3.1		1.09	0.31		10	6								
1090220	0925	3.0		0.84	0.26		16	6								
1090221	0914									90	84					
1090224	0947	3.0		0.7			14	8								
1090227	0931	2.8		0.57	0.19		15	6								

DATE	TIME	WBC	RBC	HGB	HCT	MCV	PLT	BLST	NEUT	BAND	SEG	LYM	MON	EOS	BAS
1090214	1840	8810	4.05	13.4	39.7	98.0	173		90.2			8.7	0.9	0.1	0.1
1090217	0905	5110	3.79	12.2	37.5	98.9	188		73.4			14.1	7.4	4.9	0.2
1090220	0852	6860	3.92	12.9	37.9	96.7	215		79.1			9.5	6.3	5.0	0.1
1090224	0855	7450	4.08	13.7	39.1	95.8	246				88.0	7.0	4.0	1.0	0.0
1090227	0901	5590	4.00	12.8	38.4	96.0	211		77.8			10.9	7.3	3.8	0.2

DATE	TIME	NA	K	CL	CA	BUN	CREAT	Mg	P	CRP	ESR	PCT	LIPASE	LACTATE
1090212	2003									0.550				
1090214	1841	141	4.3			19	1.04							
1090217	0722													6.4
1090217	0936	140	4.2			9	0.95							
1090220	0801													8.4
1090220	0925	138	4.4	105	7.7	21	0.98	1.9	2.8					
1090224	0801													9.6
1090224	0947	135	4.3	99	7.3	28	0.92	2.1	3.2					
1090227	0834													5.2
1090227	0931	137	3.9	104	7.1	18	0.91	2.3	3.2					

DATE	TIME	GLUCOSE	PAB	TRF	ZN	CU	KETONE	CK	CKMB	PT-P	PT-C	APTT-P	APTT-C	TRO-T
1090227	0912									11.6	10.1	31.9		
- 放射線報告 :檢驗項目 **:CT ABDOMEN-SURVEY** 報告日期 **:1090227**
  主 訴 :
  Result:
  Finding:
  Post-contrast CT of abdomen with IV contrast enhancement revealed:
  > ascending colon and cecum Ca, s/p right hemicolectomy, and drainage catheterization over right lower abdomen; with mild fluid and gas distension of small bowel loops with a transitional zone is about at ileum at right lower abdomen, and few fluid accumulation over bilateral paracolic gutter, and to the pelvis, suggested correlating with clinical history and checkup.
  > left pleural effusion, and with passive atelectasis of bilateral lower lung.
  > Post nasogastric intubation
  > An accessory spleen around 1.7cm is noted.
  > Several small cysts in bilateral kidneys are noted.
  > Mild fatty atrophy of the pancreas is noted.
  > no definite space-occupying lesion of liver parenchyma is identified
  > R/O benign prostatic hypertrophy.
  > No enlarged lymph nodes in the retroperitoneum, including pelvis.
  Impression:
  For details, please see the descriptions above.

特殊檢查

病理報告

- 活體組織切片病理報告 手術日期 : **109/02/14** 病理號 : **S0904872**

診斷醫師:楊敢順 報告日期:109/02/25
Pathologic diagnosis:
Colon, terminal ileum, and appendix, LAPA right hemicolectomy --- Adenocarcinoma, moderately to poorly differentiated, of cecum. Tumor invaded to mesocolic soft tissue with ileocecal valve involvement at cecal side. The appendix, bilateral cut ends and mesocolic lymph nodes (0/33) are free of tumor.
Ancillary study for diagnosis:
IHC stains for MLH1, MSH2, PMS2 and MSH6 are done on section E. MLH1 and PMS2 are lost in tumor.
Prognostic and predictive factor:
1. Tumor size: 4.8x3.5 cm.
2. Depth of invasion: Mesocolic soft tissue.
3. Histologic grade: Moderately to poorly differentiated (G2 to G3).
4. Angiolymphatic invasion: Not identified.
5. Perineural invasion: Not identified.
6. Tumor deposits (discontinuous extramural extension): Not identified.
7. Macroscopic tumor perforation: Not identified.
8. Surgical Margins:
a) Proximal Margin: Uninvolved by invasive carcinoma, distance of tumor from margin: 7.5 cm.
b) Distal Margin: Uninvolved by invasive carcinoma, distance of tumor from margin: 8.1 cm.
c) Radial or Mesenteric Margin: Uninvolved by invasive carcinoma, distance of tumor from margin: more than 2 cm.
9. Lymph node status: Uninvolved (0/33).
10. Extranodal involvement: Not applicable.
11. Tumor regression grading S/P CCRT: No known presurgical therapy.
12. Type of polyp in which invasive carcinoma arose: Not identified.
13. Additional pathologic findings: Not identified.
14. Pathological TNM Stage: pT3N0 (According to the eighth edition, American Joint Committee on Cancer Staging Guidelines for Tumors).
15. TNM descriptors: Not applicable.
Gross description:
The specimen consists of a segment of colon, 11.6 cm in length, and 6.7 cm in distal cut end circumference, and a segment of terminal ileum, 7.5 cm in length, and 3.8 cm in cut end circumference. An unremarkable appendix, 3.8 cm, is noted. A gray tan firm annular elevated and centrally ulcerative tumor, 4.8x3.5 cm, is noted, 8 cm to distal cut end and very close to ileocecal valve. On cross section, the tumor invaded to the mesocolic soft tissue with 2.5 cm to the radial margin. The appendix and both cut ends are free of tumor. Representative part for section: A) proximal cut end B) distal cut end C) appendix D) ileocecal valve E-J) tumor K) radial margin L-N) lymph nodes.
Note:
1. The interpretation of MMR panel and recommended studies are as below: Loss of nuclear expression of MLH1 and PMS2: testing for methylation of the MLH1 promoter and/or mutation of BRAF is indicated (the presence of a BRAF V600E mutation and/or MLH1 methylation suggests that the tumor is sporadic and germline evaluation is probably not indicated; absence of both MLH1 methylation and of BRAF V600E mutation suggests the possibility of Lynch syndrome, and sequencing and/or large deletion/duplication testing of germline MLH1 may be indicated). Please note: There are exceptions to the above IHC interpretations. These results should not be considered in isolation, and clinical correlation with genetic counseling is recommended to assess the need for germline testing.
2. This case has been peer reviewed by two doctors.
#T-59420_2 #M-81403_2 2 1105 S0000F
#T-C4560_2 #M-09183_2 0 1105 000000
#T-59200_2 #M-09183_2 0 1105 000000

其它

出院時情況

- 3.改門診治療

出院指示

藥名	劑量	劑量單位	用法	頻次	數量	數量單位	現況	天數	單位
$Biofermin tab	2	TAB	PO	TID	36	TAB	TH	6	天
Mopride tab 5mg	1	TAB	PO	TID	18	TAB	TH	6	天
XylmoL oint 15gm	1		TOP	BID	2		TH	1	瓶
Mag. Oxide tab	2	TAB	PO	TID	36	TAB	TH	6	天
Through tab	1	TAB	PO	QN	6	TAB	TH	6	天
Gaslan tab 40mg	1	TAB	PO	QID	24	TAB	TH	6	天

出院計劃

- 門診日期
  - date on 2020/03/10

主治醫師 2062F 蔣鋒帆 / 住院醫師 2303K 陳昳菩

正式摘要(電子簽章：000000187039206)

W52 / W52

doctorRank:B
